# Global research hotspots and trends of acupuncture regulating neuroplasticity: a bibliometric analysis from 2005 to 2024

**DOI:** 10.3389/fneur.2025.1615659

**Published:** 2025-09-22

**Authors:** Qiaoli Zhang, Fayan Wen, Wenxiu Qin, Xinghua Zhang, Hai Zhu, Fengfan Zhang

**Affiliations:** ^1^Department of Rehabilitation Medicine, The Second Affiliated Hospital of Gansu University of Traditional Chinese Medicine, Lanzhou, China; ^2^Clinical College of Traditional Chinese Medicine, Gansu University of Traditional Chinese Medicine, Lanzhou, China; ^3^The First Affiliated Hospital of Tianjin University of Traditional Chinese Medicine, Tianjin, China; ^4^Department of Rheumatology and Osteopathy, Gansu Provincial Hospital of Traditional Chinese Medicine, Lanzhou, China

**Keywords:** acupuncture, neuroplasticity, bibliometrics, visualization, hotspot, trends

## Abstract

**Background:**

Acupuncture has been widely used to relieve or cure various diseases. In this study, bibliometrics was used to comprehensively analyze the research focus and development trend of acupuncture regulating neuroplasticity, to provide new insights for future research.

**Method:**

In this study, the Science Citation Index-Expanded database in the Web of Science Core Collection was used to retrieve and download the publications about acupuncture regulating neuroplasticity from 2005 to 2024. CiteSpace, VOSviewer, “Bibliometric” software package and Microsoft Excel were used for bibliometric analysis, and the number of publications, countries/regions, institutions, authors, journals, references and keywords were analyzed.

**Results:**

A total of 264 publications, involving 905 institutions in 100 countries/regions. Overall, the publishing volume in the past 20 years has shown a steady upward trend. China has become the leading force in this research field, contributing the most high-quality publications. Guangzhou University of Chinese Medicine and Shanghai University of Traditional Chinese Medicine stand out as the most prolific institutions. Beijing University of Chinese Medicine and Harvard University have high academic influence. Among 1,586 authors, CZ Liu, XY Hua and JG Xu were identified as the most prolific authors. *Frontiers in Neuroscience* is the most published journal. In recent years, the mechanisms of acupuncture have attracted wide attention and become a hot topic.

**Conclusion:**

With the increasing research in this field, cooperation between different countries/regions, institutions and authors should be strengthened. AMPK, BDNF, brain-gut axis and brain-immune axis are the research hotspots and frontiers in this field. In the future, with the improvement of technical level, we should deepen the mechanism research, optimize clinical research and promote the standardization and individualization of acupuncture technology, which is expected to further reveal the scientific connotation of acupuncture regulation plasticity.

## 1 Introduction

Neuroplasticity usually refers to brain plasticity, which can be defined as changes in the structure, function or connection of the nervous system in response to individual experience or environmental stimuli (1). It is influenced by many factors, including age, genetic factors, individual health status, comorbidity, exercise, diet and sleep (2). Neuroplasticity involves complex multi-level processes, including molecules, synapses, electrophysiology and structural organization (3). It is a crucial mechanism for brain health, as it serves as the foundation for the nervous system’s ability to change and adapt to the ever-changing demands of human experience (4). It is a potential mechanism for cognitive and motor learning, and plays a key role in maintaining cognitive health and independence during the process of aging (4). This ability of brain self-reorganization is of great significance to the development of science, nerve recovery and medical rehabilitation (5).

Acupuncture, a traditional medical technology in China, is one of the most popular complementary and alternative medical forms in the world, which produces therapeutic effects by stimulating specific acupoints and meridians on the body surface (6). Stimulation methods are not limited to manual acupuncture, but also include electroacupuncture, ear acupuncture and laser acupuncture (7). Acupuncture has been widely used in various clinical diseases. In the past 20 years, with the development of medical technology, the research on acupuncture regulating brain plasticity has been greatly improved. Neuroplasticity is one of the key mechanisms of acupuncture (8). Modern medicine has proved that acupuncture can dilate blood vessels, improve blood circulation in the brain, and enhance the blood oxygen supply of damaged nerve tissues, thereby reducing the formation of free radicals, protecting the formation and growth of neurons and synapses, and promoting the proliferation of neural stem cells and the restoration of neural function (9). With the publication of numerous of literatures about acupuncture regulating neuroplasticity. It is particularly important to comprehensively analyze the development trends, current challenges, emerging themes and prospects in this field.

Bibliometrics is a comprehensive and fair scientific data analysis method, which is conducive to exploring the knowledge structure, development track, hotspots, trends and contributions of different researchers, institutions and countries (10). As a quantitative analysis method, bibliometrics has the advantages of analyzing a multitude of highly heterogeneous documents and showing past academic research activities and achievements objectively and intuitively, which is helpful to reduce the deviation of paper evaluation caused by human factors (11). So far, there is no bibliometrics report about acupuncture regulating neuroplasticity. It is of great significance to visually analyze the research status, hot spots and frontiers of acupuncture regulating neuroplasticity, which not only helps to comprehensively sort out the research progress in this field, but also provides reference for the future research direction and promotes the in-depth development of this field. Therefore, we have made a bibliometric analysis of acupuncture-regulated neuroplasticity, and constructed a corresponding visualization chart, aiming at summarizing the current situation, hot spots, trends and dynamic frontiers of acupuncture-regulated neuroplasticity research in the world.

## 2 Materials and methods

### 2.1 Data sources and retrieval strategies

**Figure 1:**
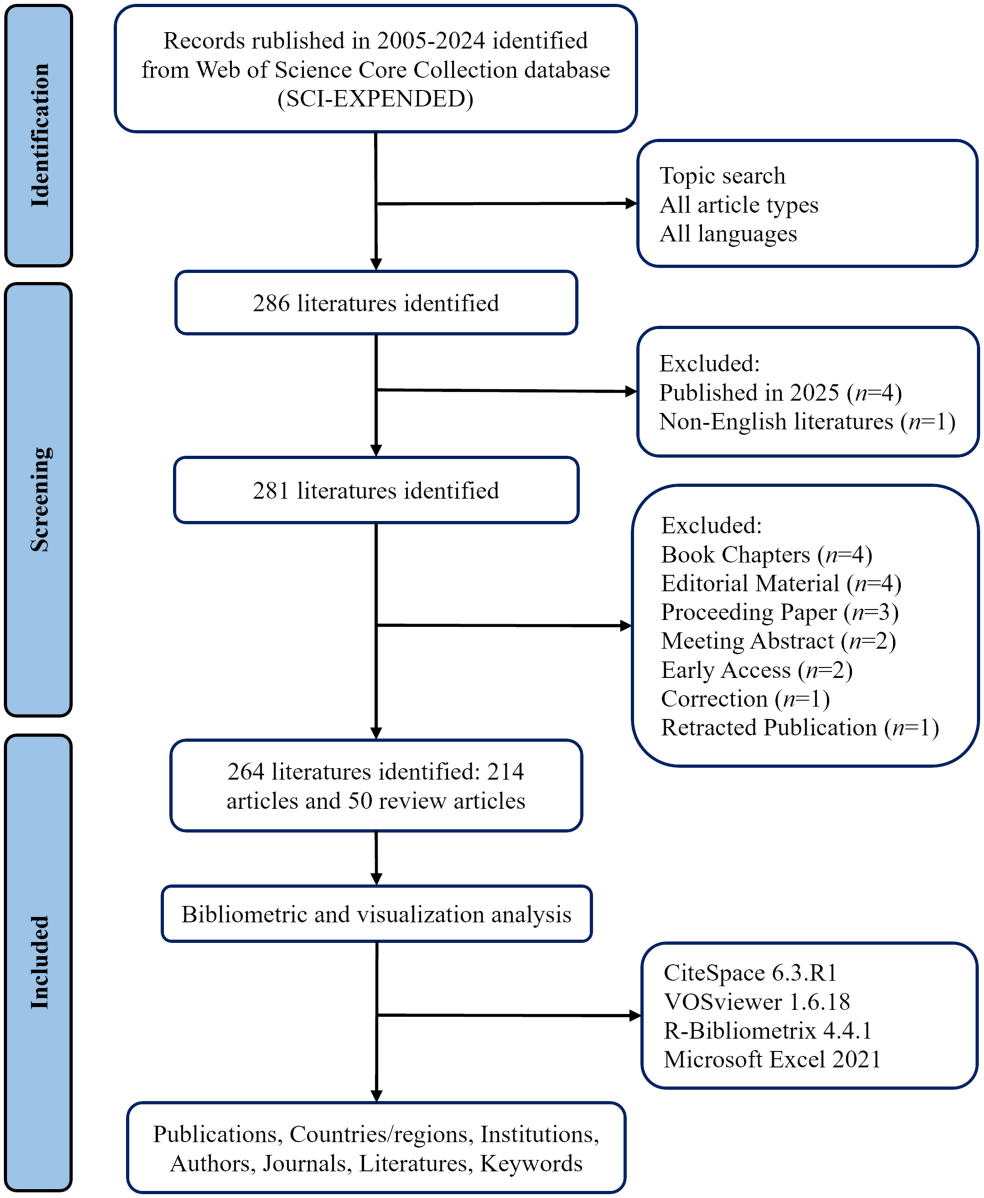
Flow diagram for the screening and analysis of publications.

Science Citation Index-Expanded (SCI-E) database has become the most sensible and widely accepted choice for bibliometrics query (12). We selected the SCI-E database in the database of the Web of Science Core Collection (WoSCC) database to search the literatures published from January 1, 2005 to December 31, 2024. The retrieval formula is shown in Table 1. All articles will be retrieved and downloaded on February 12, 2025. Regardless of language and article type, a total of 286 documents were initially retrieved. The limited language type is “English” and the article type is “article” or “review article.” Finally, a total of 264 documents were obtained, including 214 original articles and 50 review papers. Export all the included documents, including complete records and cited references. Two researchers imported them into bibliometrics tools for data analysis. If there are differences between the two researchers, they should discuss them or seek help from the third author to reach a final consensus. The flow chart of the screening process in this study is shown in Figure 1.

**Table 1:** Retrieval strategy of related research on acupuncture regulating neuroplasticity.

Retrieval condition	Retrieval formula
Records identified through the WoSCC database (SCI-E)	#1: TS = (“Acupuncture” OR “Pharmacopuncture” OR “Electroacupuncture” OR “Acupressure” OR “acupunct*” OR “Acupuncture Therapy” OR “Auricular*” OR “Acupuncture Point*” OR “needl*” OR “Ear Acupuncture” OR “Meridian*” OR “Acupoint*”) #2: TS = (“Neuroplasticity” OR “Neur* Plasticity” OR “Neur* Pruning” OR “Neuronal Remodeling” OR “Neuronal Arborization*” OR “Neuronal Network Remodeling” OR “Brain Plasticity” OR “Synaptic Plasticity” OR “Synaptic Pruning” OR “Axon* Pruning” OR “Dendrit* Pruning” OR “Dendrit* Arborization*” OR “Dendrit* Remodeling”)
Time: 2005.01.01–2024.12.31	# 3 = #1 AND #2

### 2.2 Data analysis and visualization

VOSviewer and CiteSpace are bibliometrics visualization tools, which can help to analyze the research status and detect the research frontier (13). In this study, VOSviewer (version 1.6.18) was used to analyze the cooperation network between institutions and authors, the citation of journals and the co-citation analysis of references, and the co-occurrence analysis of keywords. CiteSpace (version 6.3. R1) is used to draw the network visualization of countries/regions, the dual-map overlay of journals, the burst detection of references, keyword clustering, and the burst detection and time zone map of keywords. Bibliometrix software package (version 4.4.1) in R software is used to draw the Bradford’s law classification of Journals and the three-field plot of keywords. The annual publications are drawn by Microsoft Excel 2021. H-index of countries/regions, institutions and journals, SCIE edition, Journal Impact Factor (JIF), and Journal Citation Reports (JCR) category are obtained through WoSCC database.

## 3 Results

### 3.1 Analysis of annual publication

**Figure 2:**
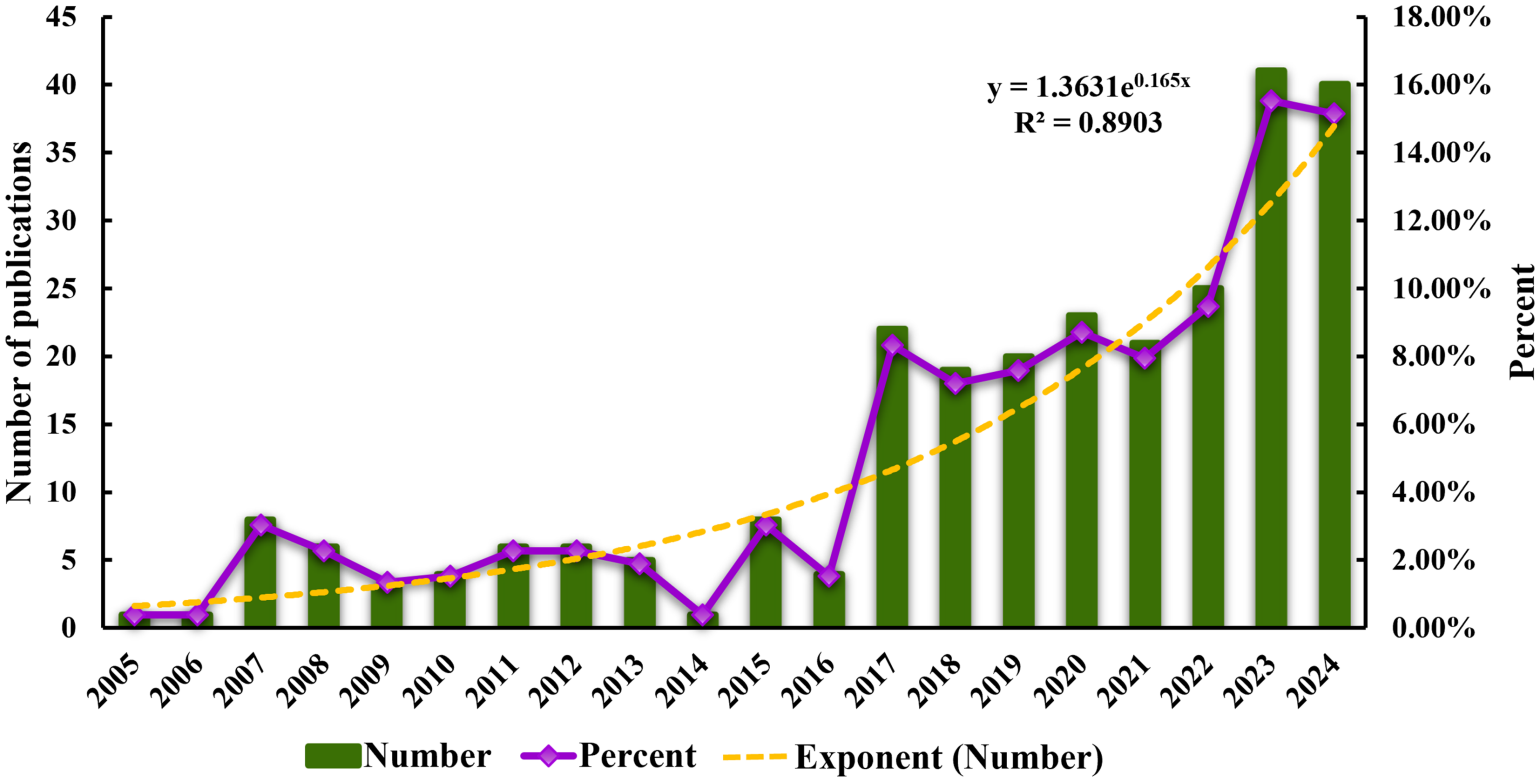
Annual publication volume and growth trend of acupuncture regulating neuroplasticity.

In the SCI-E database, a total of 264 articles on acupuncture regulating neuroplasticity published during 2005–2024 were finally included. As shown in Figure 2, about 13 papers are published every year on average. From 2005 to 2016, the number of articles published annually was relatively low. It has gradually increased since 2017, and the number of publications has increased by 450% over the previous year. However, it remained relatively stable in the next 6 years. This may be related to the global outbreak of COVID-19. In 2023, the number of published articles increased significantly, reaching the peak of 41 publications. In the past 2 years, the number of published articles accounted for 30.68% of the total number of published articles. The exponential function y = 1.3631e^0.165x^ (R^2^ = 0.8903) is obtained by drawing the fitting curve of the publication period. The closer R^2^ value is to 1, the better the fitting is, indicating that the research in this field has shown a continuous growth trend over the past 20 years. This reflects the continuous progress in this field. These trends suggest that the regulation of neural plasticity by acupuncture has received increasing academic attention and has the potential to drive the future development of this field. It is predicted that the number of published papers in this field will continue to grow in the future.

### 3.2 Analysis of countries/regions

**Figure 3:**
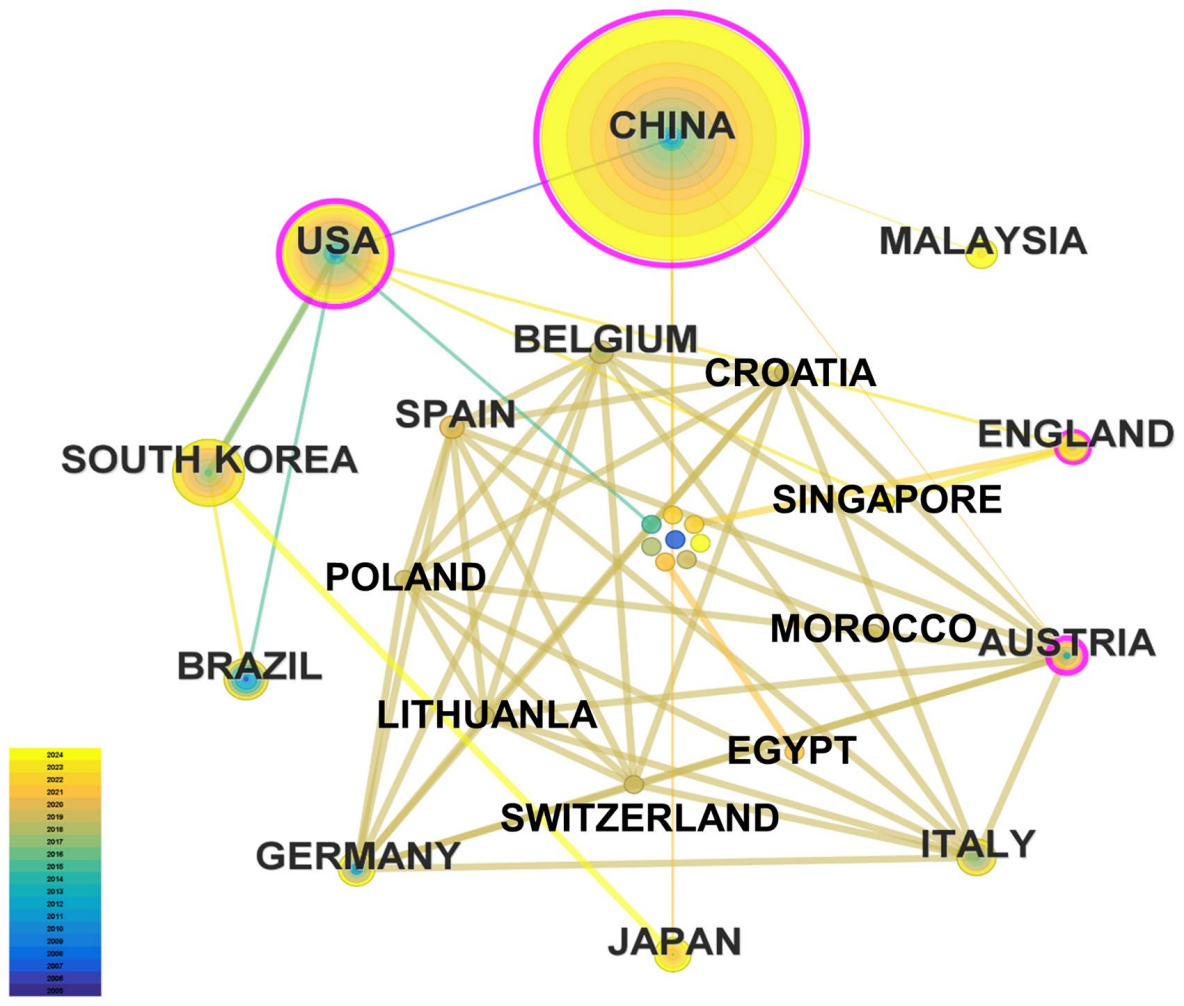
The map of collaboration network between countries/regions.

A total of 100 countries/regions participated in the publication of the research on acupuncture regulating neuroplasticity in the past 20 years. Table 2 lists the top 10 countries/regions, mainly Asian and European countries. The country that published the most papers was China (195 articles), accounting for 73.86% of the total, more than half. The USA ranks second (41 articles, 15.53%). Followed by South Korea (20 articles), Brazil (6 articles), Germany (5 articles) and Japan (5 articles). China has the highest centrality (0.67), citation frequency (2,848 times) and H-index (14). This shows that China has made great contributions to this field. Figure 3 shows the network diagram of country/region cooperation. Each node represents a country or region, and the larger the node, the more publications there are. Nodes with purple outer rings indicate high centricity (≥ 0.1). Linear links between nodes represent the degree of cooperation between countries. The map includes 27 nodes and 53 connections, and the network density is 0.151. The USA paid attention to this field earlier, and it has continued since 2005. Interestingly, although the number of papers published by Austria is relatively small, its high centricity (0.54) highlights the quality and international influence of its research. Although China has the largest number of publications in this field, the depth and breadth of its cooperation network still need to be further expanded. Overall, cooperation between countries is low.

**Table 2:** Top 10 countries/regions contributed to research publications on acupuncture regulating neuroplasticity.

Rank	Countries/ regions	Publications (%)	Centrality	Citations	Average citations	H-index
1	China (Asia)	195 (73.86)	0.67	2,848	14.61	29
2	USA (North America)	41 (15.53)	0.50	1,382	33.71	20
3	South Korea (Asia)	20 (7.58)	0.02	485	24.25	11
4	Brazil (South America)	6 (2.27)	0	79	13.17	4
5	Germany (Europe)	5 (1.89)	0	216	43.2	4
6	Japan (Asia)	5 (1.89)	0.04	85	17	4
7	Italy (Europe)	4 (1.52)	0	238	59.5	4
8	Austria (Europe)	3 (1.14)	0.54	164	54.67	2
9	England (Europe)	3 (1.14)	0.11	77	25.67	3
10	Malaysia (Asia)	3 (1.14)	0	24	8	2

### 3.3 Analysis of institution

**Figure 4:**
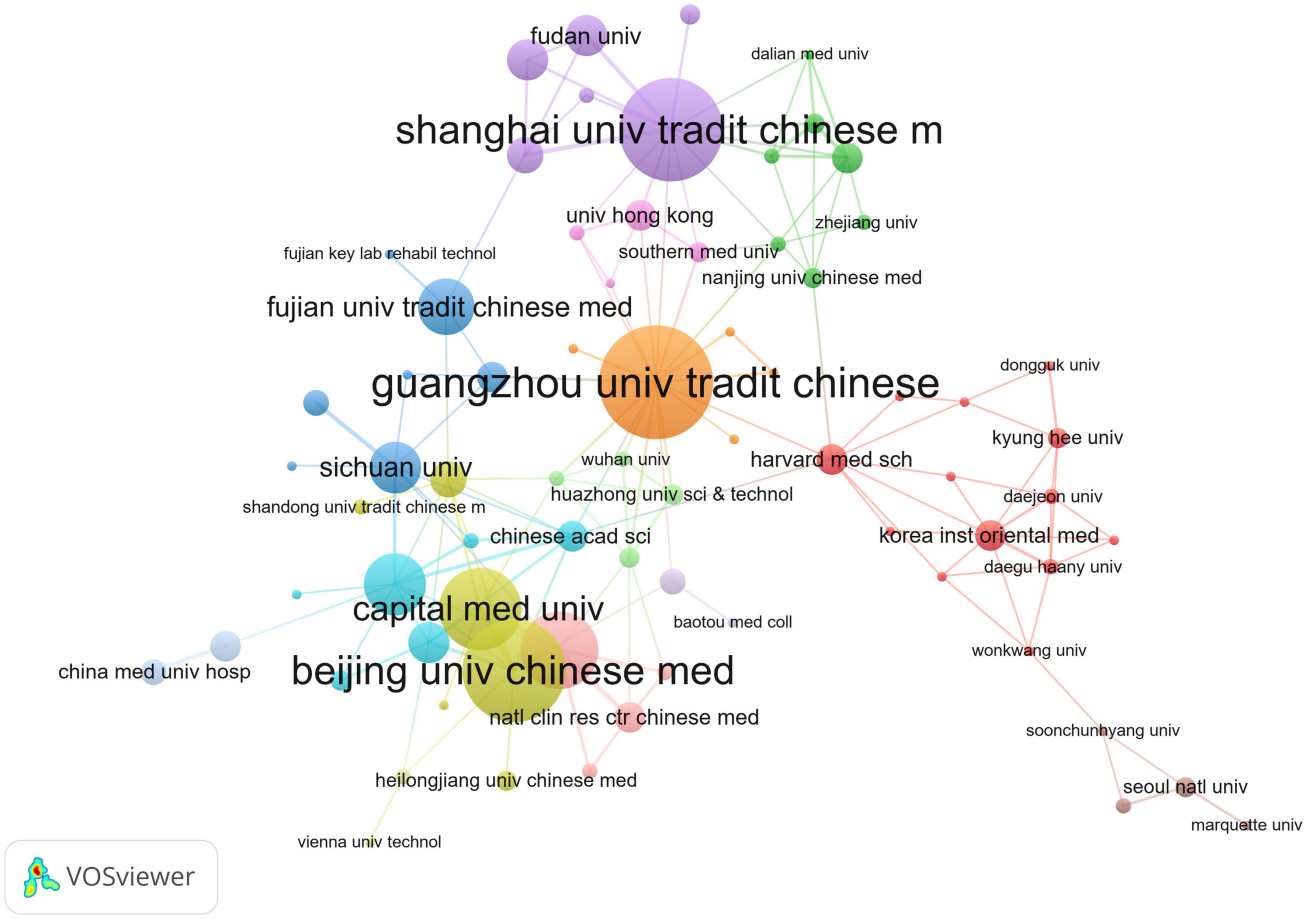
Co-occurrence map of research institutions on acupuncture regulating neuroplasticity.

In terms of research institutions, about 905 institutions have published the research results of acupuncture regulating neuroplasticity. Table 3 highlights the top ten prolific institutions in terms of published papers, mainly located in China, and concentrated in Chinese medicine higher education institutions. Guangzhou University of Chinese Medicine and Shanghai University of Traditional Chinese Medicine topped the list with 22 publications. Followed by Beijing University of Chinese Medicine (20 articles), Capital Medical University (16 articles) and Tianjin University of Traditional Chinese Medicine (15 articles). It is worth noting that Beijing University of Chinese Medicine has the highest total citation times (509 times) and H-index (13), while Harvard University has the highest average citation times (48.78 times). This shows that these two institutions have important academic influence in this research field. VOSviewer is used to analyze the cooperative relationship between institutions (Figure 4). Guangzhou University of Chinese Medicine has the closest cooperation with other institutions, and its research results will provide substantial contributions to this field in the future. The overall cooperation between institutions is relatively low.

**Table 3:** The top 10 institutions on research related of acupuncture regulating neuroplasticity.

Rank	Institutions	Publications	Citations	Average citations	H-index
1	Guangzhou University of Chinese Medicine	22	273	12.41	10
2	Shanghai University of Traditional Chinese Medicine	22	253	11.5	10
3	Beijing University of Chinese Medicine	20	509	25.45	13
4	Capital Medical University	16	403	25.19	10
5	Tianjin University of Traditional Chinese Medicine	15	294	19.6	8
6	Chengdu University of Traditional Chinese Medicine	13	247	19	7
7	Fujian University of Traditional Chinese Medicine	11	148	13.45	7
8	Sichuan University	10	164	16.4	6
9	China Academy of Chinese Medical Sciences	9	157	17.44	7
10	Harvard University	9	439	48.78	7

### 3.4 Analysis of author

**Figure 5:**
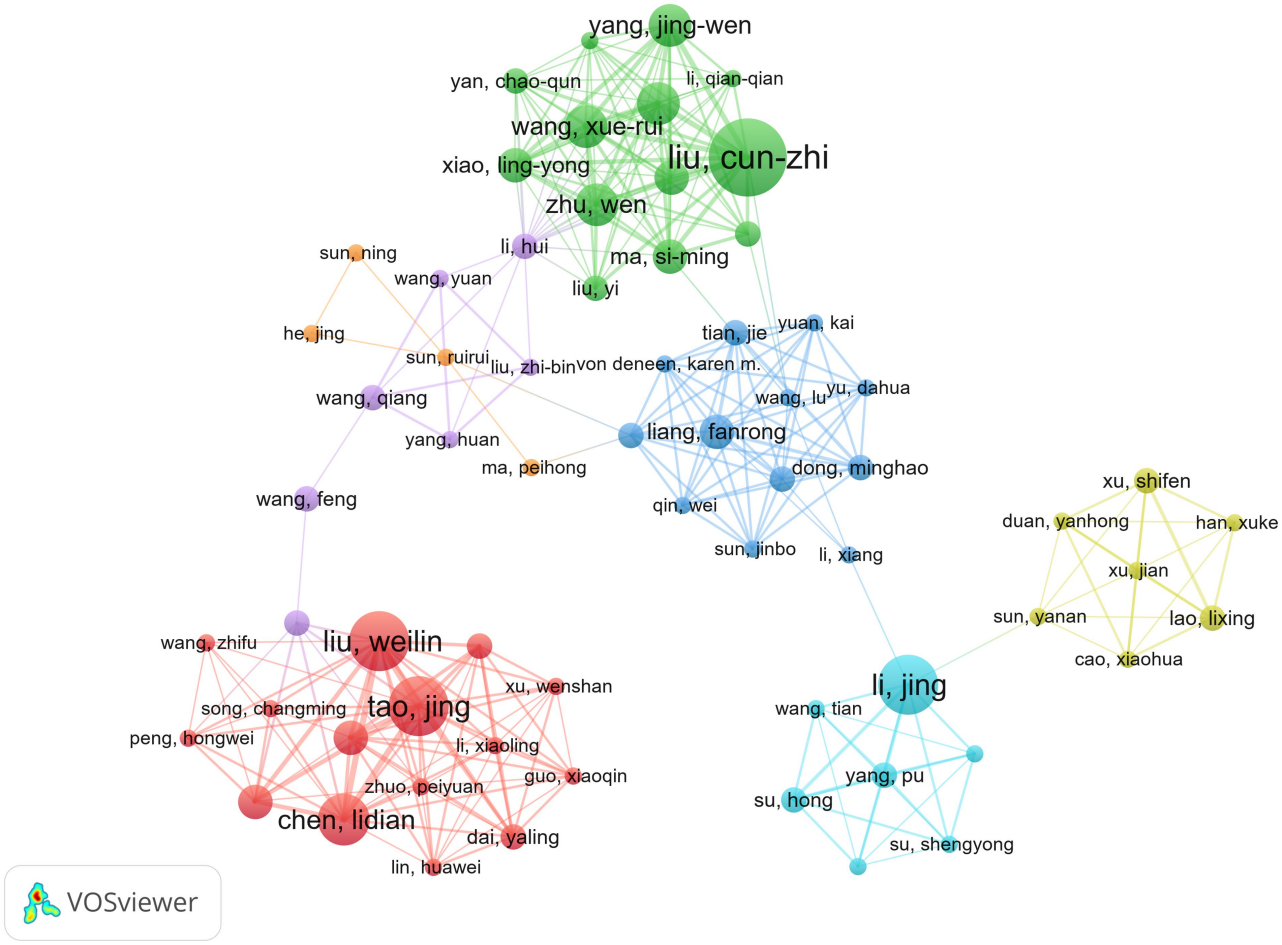
Author collaboration network diagram of acupuncture regulating neuroplasticity.

Over the past two decades, about 1,586 authors participated in the publication of these 264 documents. To fully show the influence of important authors, the number of publications and citation weight are used to represent (15). Among the top 10 authors, as shown in Table 4. CZ Liu from Beijing University of Chinese Medicine leads with 9 publications and 398 citations. XY Hua and JG Xu are also the most published authors. This highlights their great contribution to this field and emphasizes the wide recognition of their research in academic circles. The cooperative relationship between authors is shown in Figure 5. These authors are scattered and have not formed a large cooperative team. The cooperation between authors in different institutions is low.

**Table 4:** Top 10 authors contributed to the research related to acupuncture regulating neuroplasticity.

Rank	Authors	Publications	Citations	Institution
1	Liu CZ	9	398	Beijing University of Chinese Medicine
2	Hua XY	9	54	Yueyang Hospital of Integrated Traditional Chinese and Western Medicine
3	Xu JG	9	54	Yueyang Hospital of Integrated Traditional Chinese and Western Medicine
4	Wu JJ	8	41	Yueyang Hospital of Integrated Traditional Chinese and Western Medicine
5	Liu WL	7	108	Fujian University of Traditional Chinese Medicine
6	Tao J	7	108	Fujian University of Traditional Chinese Medicine
7	Li J	7	84	Guangxi University of Chinese Medicine
8	Chen LD	6	99	Fujian University of Traditional Chinese Medicine
9	Zheng MX	6	18	Yueyang Hospital of Integrated Traditional Chinese and Western Medicine
10	Wang TH	6	128	West China Hospital

### 3.5 Journal publications and citation analysis

**Figure 6:**
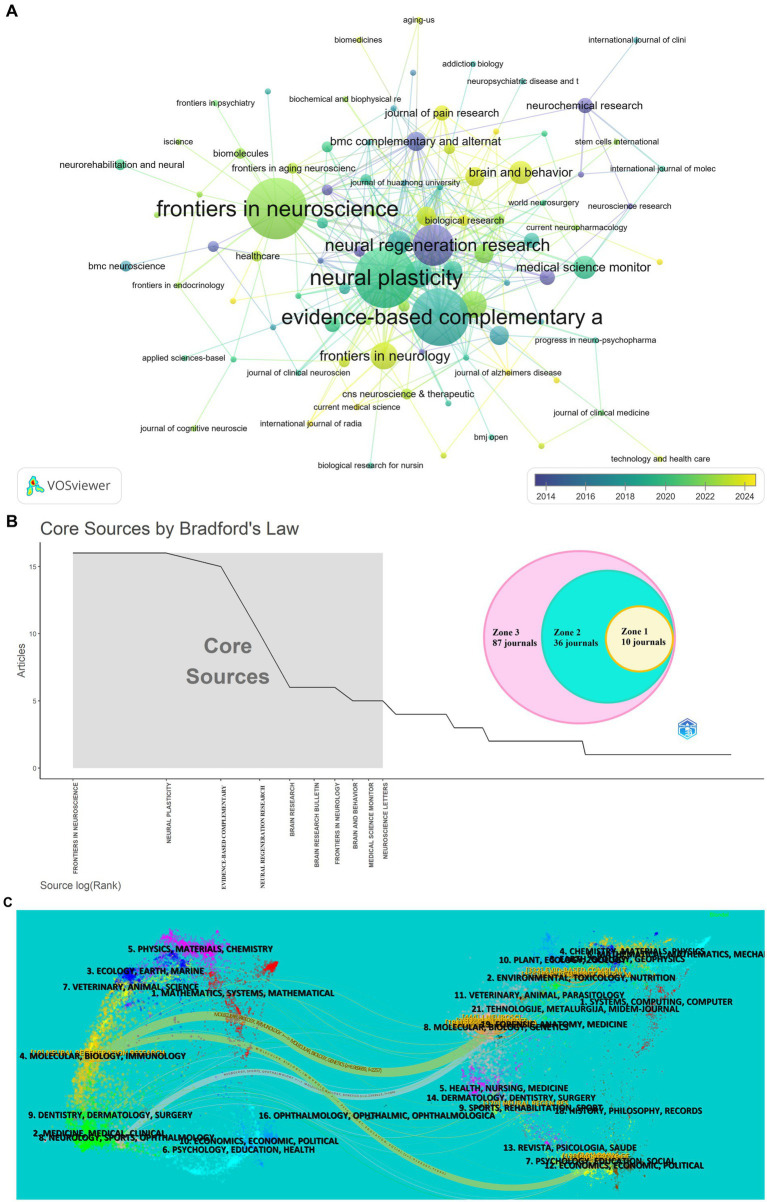
Journal analysis of acupuncture regulating neuroplasticity research. **(A)** Co-occurrence map of journals. **(B)** Classification of journals according to Bradford’s law. **(C)** Dual-map overlay of journals.

The publications about acupuncture regulating neuroplasticity are published in 133 journals. Table 5 shows the top 10 most influential journals, mainly related to neuroscience. These journals published 90 articles, accounting for 34.09% of all publications in this field. The two core journals with the largest number of articles are *Frontiers in Neuroscience* and *Neural Plasticity* respectively, with 16 articles published each (6.06%). It is worth noting that the number of citations (360 times), the average number of citations (22.5 times) and the H-index (13) of the journal *Neural Plasticity* are all the highest, indicating that the published literatures are of high quality. 90% of these 10 journals are from the USA and Europe, which shows that they have played a vital role in promoting academics. However, the JIF of these 10 journals is relatively low, and JCR is mostly located in Q2/Q3. As shown in Figure 6A, *Neural Regeneration Research*, *Neurochemical Research*, and *BMC Complementarity and Alternative Medicine* paid attention to this field earlier, while *Frontiers in Neurology* and *Brain and Behavior* have shown great interest in this field in recent years. Figure 6B shows that journals are divided into three regions based on Bradford’s law. Region 1 contains 10 core journals, Region 2 covers 36 secondary journals, and Region 3 includes 87 peripheral journals.

**Table 5:** Top 10 journals on research related to acupuncture regulating neuroplasticity.

Rank	Journal	Publications (%)	Citations	Average citations	SCIE edition	Country/region	JIF (2023)	JCR (2023)	H-index
1	Frontiers in Neuroscience	16 (6.06)	309	19.31	Neurosciences	Switzerland	3.2	Q2	7
2	Neural Plasticity	16 (6.06)	360	22.5	Neurosciences	USA	3	Q2	13
3	Evidence Based Complementary and Alternative Medicine	15 (5.68)	226	15.07	Integrative & Complementary Medicine	USA	/	Q3	9
4	Neural Regeneration Research	10 (3.79)	111	11.1	Cell Biology / Neurosciences	China	5.9	Q2, Q1	5
5	Brain Research	6 (2.27)	95	15.83	Neurosciences	Netherlands	2.7	Q3	4
6	Brain Research Bulletin	6 (2.27)	80	13.33	Neurosciences	USA	3.5	Q2	4
7	Frontiers in Neurology	6 (2.27)	31	5.17	Clinical Neurology/Neurosciences	Switzerland	2.7	Q2, Q3	3
8	Brain and Behavior	5 (1.89)	20	4	Behavioral Sciences/ Neurosciences	USA	2.6	Q2, Q3	3
9	Medical Science Monitor	5 (1.89)	66	13.2	Medicine, Research & Experimental	USA	2.2	Q3	4
10	Neuroscience Letters	5 (1.89)	70	14	Neurosciences	Netherlands	2.5	Q3	4

Figure 6C shows a dual-map overlay of related journals on acupuncture regulation of neuroplasticity. The citing journals are displayed on the left side, and the cited journals are on the right side. The color path shows the citation relationship. There are three main citation paths in the diagram. Papers published in Molecular/Biology/Immunology usually cite papers published in Molecular/Biology/Genetics (*z* = 6.970, *f* = 2,257) and Psychology/Education/Social (*z* = 2.237, *f* = 794). Most of the literatures published in Neurology/Sports/Ophthalmology quoted papers published in Molecular/Biology/Genetics (*z* = 1.917, *f* = 695).

### 3.6 Analysis of references

**Figure 7:**
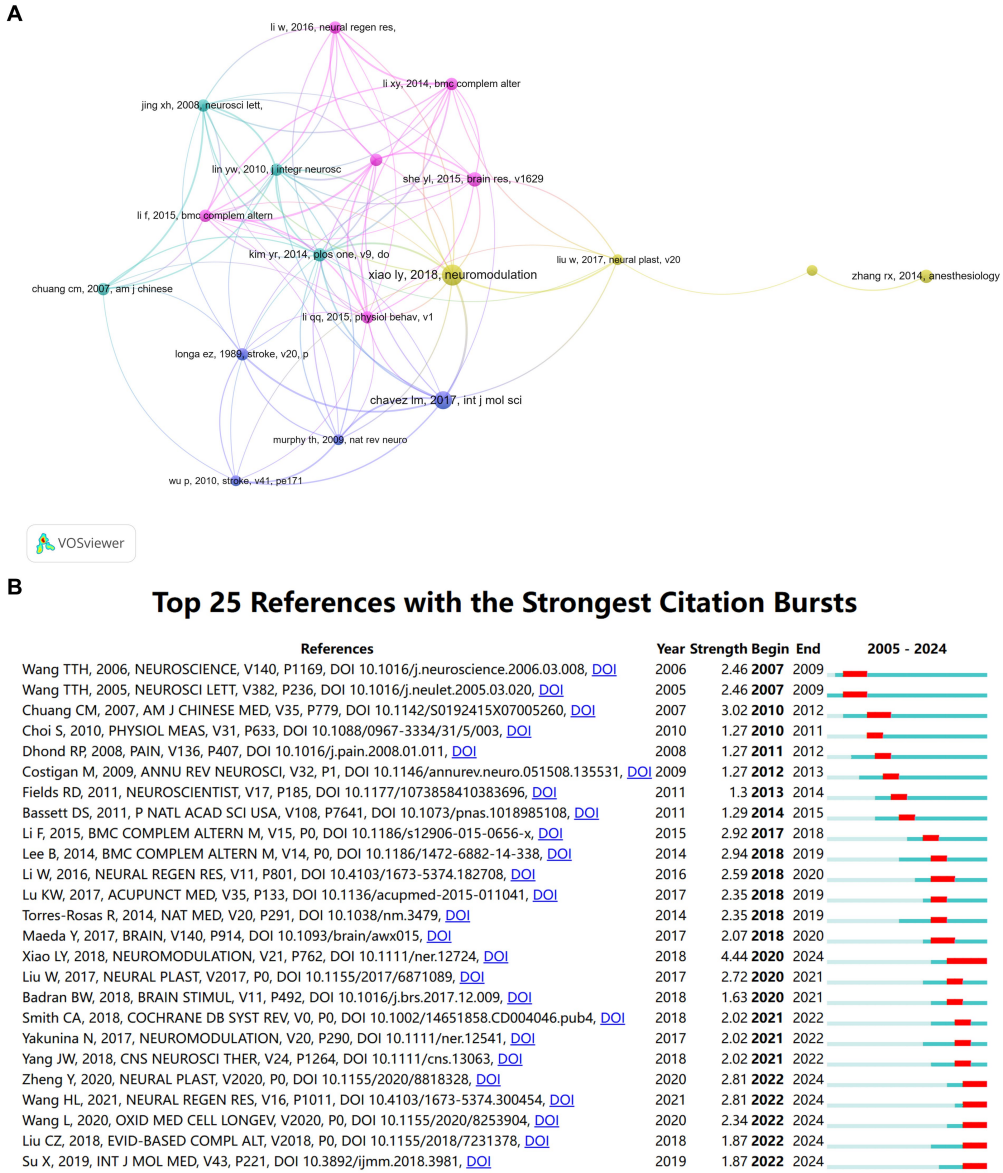
Reference analysis of acupuncture regulating neuroplasticity research. **(A)** Visual network diagram of the top 18 references co-cited references. **(B)** Reference burst detection of the top 25 references with the strongest emergent strength.

Co-cited references, including multiple references cited together in one or more publications, are beyond the search period of this study and represent a wide range of public information resources in this field (16). In this study, VOSviewer is used to analyze the co-cited references to determine the most influential ones. There are 15,144 references in 264 publications. The top 14 co-cited references are shown in Table 6, including 11 articles and 3 reviews. Six of them were published in high-impact journals. The most frequently co-cited reference is an article published in *Neuromodulation* by LY Xiao team in 2018. This study reviews the preclinical evidence and related mechanisms of acupuncture in regulating neuroplasticity, indicating that neuroplasticity may be a potential bridge between acupuncture and the treatment of various neurological diseases. Another widely cited article, written by LM Chavez in 2017, was published in the *International Journal of Molecular Sciences*. This study summarized the known mechanisms of acupuncture and electroacupuncture for the rehabilitation of ischemic stroke at that time, and pointed out that DU20 (Baihui), ST36 (Zusanli), LI11 (Quchi), DU26 (Shuigou), DU14 (Dazhui) and LI4 (Hegu) were commonly used acupuncture points involved in these effects. Figure 7A presents a visual atlas of 18 references co-cited at least 10 times in the study of acupuncture regulating neuroplasticity in the past two decades, which provides convenient access to numerous of practical information for future researchers.

**Table 6:** Top 14 co-cited references and related information on research related to acupuncture regulating neuroplasticity.

Rank	Title	First author	Year	Citations	Journal	JIF (2023)	Type
1	Applications of acupuncture therapy in modulating plasticity of central nervous system	Xiao LY	2018	19	Neuromodulation	3.2	Review
2	Mechanisms of acupuncture therapy in ischemic stroke rehabilitation: a literature review of basic studies	Chavez LM	2017	16	International Journal of Molecular Sciences	4.9	Review
3	Possible antidepressant effects and mechanism of electroacupuncture in behaviors and hippocampal synaptic plasticity in a depression rat model	She YL	2015	13	Brain Research	2.7	Article
4	Electroacupuncture promotes post-stroke functional recovery via enhancing endogenous neurogenesis in mouse focal cerebral ischemia	Kim YR	2014	12	PLoS One	2.9	Article
5	Mechanisms of acupuncture-electroacupuncture on persistent pain	Zhang RX	2014	12	Anesthesiology	9.3	Review
6	Acupuncture stimulation at Baihui acupoint reduced cerebral infarct and increased dopamine levels in chronic cerebral hypoperfusion and ischemia–reperfusion injured sprague–dawley rats	Chuang CM	2007	11	The American Journal of Chinese Medicine	4.8	Article
7	Electroacupuncture restores learning and memory impairment induced by both diabetes mellitus and cerebral ischemia in rats	Jing XH	2008	11	Neuroscience Letters	2.5	Article
8	Acupuncture attenuates cognitive deficits and increases pyramidal neuron number in hippocampal CA1 area of vascular dementia rats	Li F	2015	11	BMC Complementary and Alternative Medicine	——	Article
9	Hippocampal cAMP/PKA/CREB is required for neuroprotective effect of acupuncture	Li QQ	2015	11	Physiology & Behavior	2.4	Article
10	High-frequency electroacupuncture evidently reinforces hippocampal synaptic transmission in Alzheimer’s disease rats	Li W	2016	11	Neural Regeneration Research	5.9	Article
11	Electroacupuncture decreases cognitive impairment and promotes neurogenesis in the APP/PS1 transgenic mice	Li XY	2014	11	BMC Complementary and Alternative Medicine	——	Article
12	Electroacupuncture at Baihui acupoint (GV20) reverses behavior deficit and long-term potentiation through N-methyl-d-aspartate and transient receptor potential vanilloid subtype 1 receptors in middle cerebral artery occlusion rats	Lin YW	2010	11	Journal of Integrative Neuroscience	2.5	Article
13	Reversible middle cerebral artery occlusion without craniectomy in rats	Longa EZ	1989	11	Stroke	7.9	Article
14	Acupuncture Attenuated Vascular Dementia-Induced Hippocampal Long-Term Potentiation Impairments via Activation of D1/D5 Receptors	Ye Y	2017	11	Stroke	7.9	Article

Burst detection of references is used to identify research frontiers and emerging references (17). CiteSpace is used to analyze the strongest citation burst of references (Figure 7B). The reference “DOI:10.1111/ner.12724” (18) is the document with the strongest burst (strength = 4.44) and has been frequently cited since 2020. In addition, the documents “DOI:10.1155/2020/8818328” (19), “DOI:10.4103/1673-5374.300454” (20), “DOI:10.1155/2020/8253904” (21), “DOI:10.1155/2018/7231378” (22) and “DOI:10.3892/ijmm.2018.3981” (23) are also the most urgent references in recent 3 years.

### 3.7 Keywords analysis

**Figure 8:**
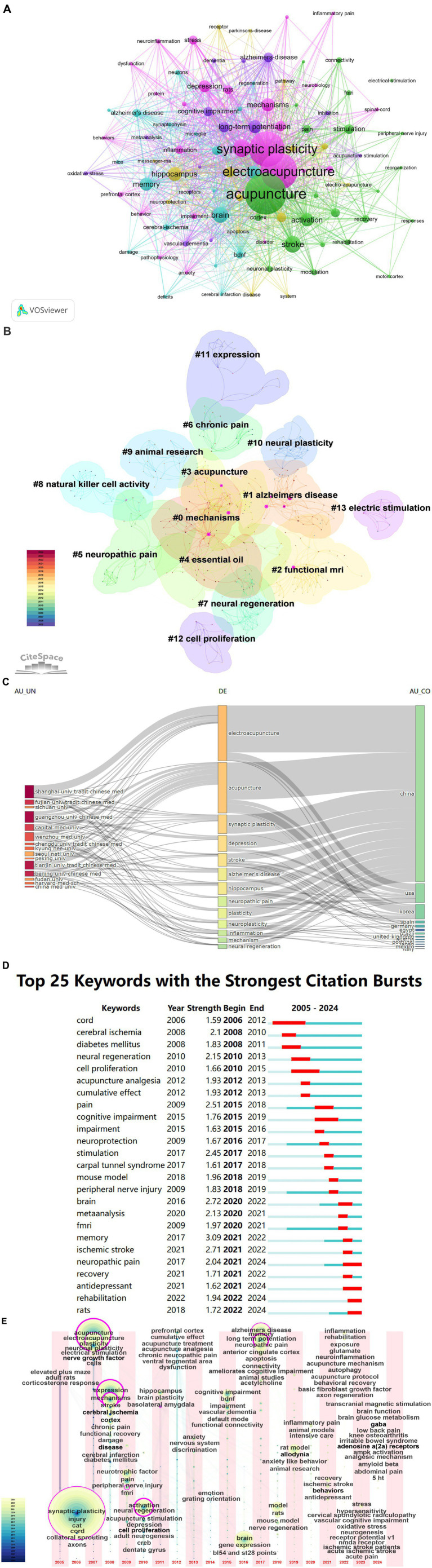
Keyword analysis of acupuncture regulating neuroplasticity research. **(A)** Visual network diagram of keywords. **(B)** Cluster analysis of keywords. **(C)** Three-field plot of the keywords. **(D)** Keyword burst detection of the top 25 references with the strongest emergent strength. **(E)** The time zone map of keywords.

Keywords represent the core content and research theme of the literature (24). In this study a total of 1,586 keywords were extracted from publications using VOSviewer. “Acupuncture” is the core of all keywords with the highest frequency (109 times) and the widest association with other keywords (strength = 586). Other frequently-occurring keywords include “electroacupuncture” (94 times) “synaptic plasticity” (90 times) “expression” (39 times) “stroke” (36 times) “brain” (34 times) and “hippocampus” (29 times) (Figure 8A). A total of 14 clusters were generated by keyword cluster analysis: #0 mechanisms #1 Alzheimer’s disease #2 functional mir #3 acupuncture #4 essential oil #5 neuropathic pain #6 chronic pain #7 neural regeneration #8 natural killer cell activity #9 animal research #10 neural plasticity #11 expression #12 cell proliferation and #13 electric stimulation. It indicates that these clusters are prominent research topics and have attracted a lot of scholars’ attention in recent years (Figure 8B).

Figure 8C further shows the proportion of core themes of each institution and country. As can be seen from the figure, almost all institutions and countries have contributed to the 13 topics represented by keywords. However, there are differences. In terms of institutions, Guangzhou University of Chinese Medicine is more interested in “depression.” Wenzhou Medical University pays great attention to “inflammation.” Seoul National University and Tianjin University of Traditional Chinese Medicine are more focused on “Alzheimer’s disease.” Beijing University of Chinese Medicine is more involved in “stroke.” As far as the country is concerned, China has made great contributions to all these hot spots. The USA focuses on “depression,” “neuropathic pain” and “stroke.” Korea is more involved in “hippocampus.”

The strongest bursts of keywords can clarify the research hotspots and trends in specific fields (25). Figure 8D shows the top 25 keywords with the strongest bursts in chronological order. The keywords of the first five bursts are “memory” (strength = 3.09), “brain” (strength = 2.72), “ischemic stroke” (strength = 2.71), “pain” (strength = 2.51) and “stimulation” (strength = 2.45). Recently, the research focuses on the key words “neuropathic pain,” “antidepressant,” “rehabilitation” and “rats.”

Figure 8E dynamically reflects the changing trend of hot spots in the research field of acupuncture regulating neuroplasticity with time. As can be seen from the figure, the key words in the period between 2005 and 2010 mainly focused on molecular signal pathways (such as glutamatergic system) and brain region specificity (such as prefrontal cortex and hippocampus). Mainly led by animal models, these papers explored the direct influence of acupuncture on neuron survival and synaptic transmission, which embodies the regulation mechanism of acupuncture on basic nerve plasticity. During the period of 2011–2018, the research focus turned to clinical major nervous system diseases, such as stroke, cerebral ischemia, Alzheimer’s disease and cognitive impairment, and functional magnetic resonance imaging (fMRI) was used to reveal the effect of acupuncture on the dynamic remodeling of brain network. During this period, the animal model studied explosive growth, focusing on the regulatory effect of acupuncture on functional recovery after cerebral ischemia and central sensitization of chronic pain. In 2019–2024, the research focus went deep into the molecular mechanism [such as brain-derived neurotrophic factor (BDNF) pathway and AMP-activated protein kinase (AMPK)] and extended to the field of metabolism-nerve intersection (such as diabetic neuropathy and anxiety comorbidity). The research scope breaks through the framework of single disease, pays attention to the systematic regulation of acupuncture on “brain-intestine axis” and “brain-immune axis,” and integrates molecular imaging, gene coding and clinical omics data by technical means.

## 4 Discussion

### 4.1 General information

According to the results of SCI-E database search, there are 264 articles about acupuncture regulating neuroplasticity in the past 20 years. The analysis of the annual publishing trend shows that the total number of global publications related to domain research has shown an overall growth trend, especially since 2023. This shows that the research in this field has entered a stage of rapid development and maintained a high degree of enthusiasm. It is predicted that the output rate of related literature in this field will continue to increase in the future. Among the 100 countries/regions participating in the research in this field, China is dominant, and the amount of published articles accounts for a large part of the total publications. This reflects the profound cultural and historical origin of China and acupuncture, and the strong support of the government and institutions for the research of traditional Chinese medicine. Compared with China, other countries, such as USA, South Korea and Brazil, have made remarkable contributions to this field, although their publications are few. In addition, Austria highlights the quality and international influence of its research with high centrality. These geographical distributions indicate the global interest in the field of acupuncture regulating neuroplasticity. It is worth noting that although China has the largest number of publications in this field, its cooperation with other countries is not frequent. China should continue to increase financial input and promote international cooperation and exchanges.

As far as research institutions are concerned, the top 10 institutions are mainly from China, which further highlights China’s important contribution to this research field. Guangzhou University of Chinese Medicine and Shanghai University of Traditional Chinese Medicine have made great contributions in this field. Beijing University of Chinese Medicine has the highest total citation times and H-index, which shows that it has high academic quality and influence. In addition, as a world-renowned university with high influence, Harvard University has the highest average number of citations, which indicates the growing interest and investment in acupuncture to regulate neuroplasticity in the USA. As far as the research authors are concerned, the key figures include CZ Liu, XY Hua and JG Xu. They have published many papers with the greatest influence, and their role in this field is crucial enough for scholars to refer to their work. However, the level of cooperation between different authors is low. Based on these findings, researchers from different countries/regions and institutions should strengthen cooperation, reduce academic obstacles, achieve high-quality research, and promote the development of research in the field of acupuncture regulating neuroplasticity.

In this study, a total of 133 journals were identified. The top 10 journals have published more than one third of the literatures, which are important journals in this field. These articles are mainly published in professional journals related to neuroscience. It can be inferred that the hotspots and trends of future research in this field may be displayed in these journals. *Frontiers in Neuroscience* and *Neural Plasticity* are the most popular journals, ranking first in the number of published articles. It shows that they have high academic status and international influence in this field. However, the overall JIF of the top 10 journals is low. One potential reason is that acupuncture research is highly specialized, which may not attract a wider and more common audience of high-impact journals. Many top journals tend to focus on research with wide applicability in different medical fields, while acupuncture research usually focuses on specific treatment methods or patient groups, which limits its fit with more versatile and influential journals. Therefore, researchers should strengthen research design and provide high-quality papers for publication in high-impact academic journals. Although challenges still exist, acupuncture research’s stable existence in professional journals has laid a solid foundation for future growth and wide acceptance of high-impact publications.

### 4.2 Research hotspots and frontiers

Through the analysis of references and keywords, we identified the main hot areas of acupuncture regulating neuroplasticity at present, and put forward the potential direction of future research. We found that the mechanism pathway of acupuncture regulating neuroplasticity is the hotspots and frontier of current research.

As a supplementary medical method, acupuncture is very popular all over the world, and has been used for various nervous system diseases, heart diseases, neuropathic pain, cancer, asthma, polycystic ovary syndrome and psychological disorders (26). In the past decades, the basic and clinical research of acupuncture has made great progress. Scientists try to explore the physiological and biological mechanism of acupuncture. In recent years, due to the rapid development of biotechnology, a series of breakthroughs have been made in the understanding of acupuncture regulation mechanism. Some basic studies show that acupuncture has many functions, such as analgesia, muscle relaxation, anti-inflammatory, mild anti-anxiety and anti-depression, and its possible biological mechanisms include central sensitization, neurotransmitters, intestinal flora, immune regulation, oxidative stress and neuroinflammation (27). In this study, we found that the following four pathways are the hotspots and frontiers in this field.

#### 4.2.1 AMPK

AMPK is the main sensor of cellular energy at adenine nucleotide level, which evolves and maintains in all eukaryotes, and feels the energy content of cells through direct interaction with adenosine triphosphate, adenosine diphosphate and adenosine monophosphate (28). The activation of AMPK is related to the regulation of glucose, lipid and protein metabolism and the process of cell adaptation (such as autophagy and mitochondrial remodeling), and the activated AMPK plays a role by phosphorylating downstream effectors (29). It is reported that AMPK disorder exists in neurodegenerative diseases, such as stroke, Alzheimer’s disease, Parkinson’s disease, Huntington’s disease, multiple sclerosis and other neurological diseases (14, 30, 31). AMPK activation seems to have both neuroprotective and apoptosis-promoting effects, which may depend on the type of nerve cells, the nature of injury, and the intensity and duration of AMPK activation (32).

More and more evidence show that acupuncture can exert a wide range of neuroprotective effects by inhibiting neuronal apoptosis (33). Guo et al. (34) found that acupuncture intervention can reduce neuronal apoptosis by promoting energy metabolism and mitochondrial biosynthesis in the brain, and then alleviate the progress of ischemic stroke in rats. The mechanism may be mediated by inducing AMPK/peroxisome proliferator-activated receptor gamma coactivator 1-alpha (PGC-1α) axis, in which AMPK is the therapeutic target. Cui et al. (35) found that the analgesic mechanism induced by electroacupuncture may be helpful to promote the autophagy of spinal microglia mediated by AMPK/mammalian target of rapamycin. Electroacupuncture can reduce the expression of p62 and increase the expression of Beclin-1 and Light chain 3-II/Light chain 3-I, which provides a potential therapeutic target for neuropathic pain. Guo et al. (36) found that electroacupuncture combined with sulforaphane can alleviate the skeletal muscle morphology and function of mice with sarcopenia by activating AMPK/sirtuin 1/PGC-1α pathway to mediate the repair of mitochondrial damage. Ding et al. (37) found that electroacupuncture can improve the depression-like behavior of post-stroke depression rats by activating AMPK and promoting mitochondrial function. Lan et al. (38) found that electroacupuncture activated AMPK through cannabinoid type 2 (CB2) receptors, and enhanced the expression of *β*-endorphin in mice inflamed skin, then to relieve inflammatory pain. This study revealed the new relationship between endocannabinoids, endorphins and AMPK in electroacupuncture analgesia, and highlighted CB2 receptors-AMPK-β-endorphin. Lyu et al. (39) found that electroacupuncture could improve the depressive behavior of ovariectomy rats by regulating synaptic plasticity and activating AMPK/nuclear respiratory factor-1/PGC-1α/mitochondrial transcription factor a signal pathway. In a word, acupuncture activating AMPK to regulate neuroplasticity is related to acupuncture regulating energy metabolism, inhibiting apoptosis, promoting neurotransmitter release and regulating synaptic function.

#### 4.2.2 BDNF

BDNF is a member of neurotrophic protein family, which plays an important role in neurogenesis, growth, differentiation, survival and plasticity of neural network, making it have an impact on various neurological and mental diseases (40, 41). It has been proved that the decrease of BDNF level may be related to a series of pathology and neuron death in the field of neurodegeneration (42). Including Alzheimer’s disease, Parkinson’s disease, Huntington’s disease and amyotrophic lateral sclerosis, depression and anxiety (43). Many studies have confirmed that acupuncture therapy is beneficial to regulate the BDNF level. It is found that acupuncture treatment lasting for 21 days can improve fear memory, cognitive function and spatial memory of patients with post-traumatic stress disorder by regulating neuroinflammation in the brain and BDNF mRNA expression in hippocampus (44). In cancer survivors with insomnia and low baseline BDNF, acupuncture can significantly increase the serum BDNF level (45). Another study found that electroacupuncture can alleviate chronic inflammatory pain and related depressive behaviors, protect hippocampal neuron structure from damage, and regulate the levels of 5-hydroxytryptamine/gamma-aminobutyric acid (GABA)/Glu in hippocampus, and significantly increase the expression of synaptic related proteins such as postsynaptic density protein-95 and synuclein by activating BDNF/tyrosine-protein kinase B/cyclic adenosine monophosphate response element binding protein (46).

In recent years, researchers have paid attention to the study of BDNF mechanism of acupuncture on patients with depression. Acupuncture is a promising non-drug therapy for relieving depressive symptoms, which can replace drug therapy or supplementary therapy to improve the results (47). The pathogenesis of depression and the potential mechanism of antidepressant treatment are related to the BDNF/tissue plasminogen activator (tPA) cleavage pathway (48). It is found that electroacupuncture can reverse the depression-like behavior of rats with unpredictable mild stress-induced depression, which may be related to BDNF pathway in hippocampus. Electroacupuncture can up-regulate the levels of tPA, BDNF, tropomyosin receptor kinase B and BDNF mRNA in hippocampus and the content of tPA mRNA in raphe nuclei of rats (49). In patients with diabetes and depression, acupuncture may increase the expression of BDNF and improve the depressive symptoms and glycemic control of patients (50). In one study, rats with stress symptoms of social failure were treated with acupuncture at GV20 (Baihui) and Ex-HN3 (Yintang) points for 2 weeks, and it was found that acupuncture could restore the decreased expressions of BDNF, neurotrophin (NT)-3 and NT-4/5 in rats, effectively correct the imbalance of NTs expression, and induce the expression of NTs (51). This study also found that the effect of acupuncture on the expression of NTs appeared earlier than that of antidepressants. These indicate that acupuncture is an effective treatment for depression, and its induced BDNF expression can effectively improve mood.

#### 4.2.3 Brain-intestine axis

As the peripheral tissue with the densest nerve distribution in the body, the intestine has many ways to establish a bidirectional “brain-intestine axis” with the central nervous system (CNS) (52). The brain-intestine axis is the main connection between digestive tract and CNS, which is composed of neural pathways such as the enteric nervous system, the sympathetic and spinal vagus nerve, and the humoral pathways involving cytokines, hormones and neuropeptides (53). The intestine sends signals to the brain through the spinal and vagus visceral afferent pathways and receives sympathetic and parasympathetic inputs through the vagus nerve, from the brain to the abdominal cavity, and contains motor, sensory and autonomic nerve fibers, which affect human development and behavior (54). Intestinal microbiota will produce neurotransmitters, such as GABA, histamine, dopamine, norepinephrine and serotonin, and other neuroactive molecules (55). The enteric nervous system is the internal nervous system of gastrointestinal tract, in which neurons organized into microarrays can regulate gastrointestinal function independently of CNS. However, although these systems are interconnected and interact with each other, preclinical and human studies show that intestinal microflora is involved in the regulation of social behaviors in a complicated way, such as depressive behaviors, physical manifestations and motivations, and more and more people realize the role of intestinal microflora in neurodegenerative diseases (56).

Related research has confirmed that acupuncture can adjust the disorder of intestinal microflora and restore the balance in the body by interfering with all aspects of the brain-intestine axis (57). Acupuncture can affect the abundance and structure of intestinal bacteria and balance the number and proportion of probiotics and pathogenic bacteria in the host. Conversely, acupuncture can reverse the metabolic disorder of various intestinal flora caused by various diseases by restoring the functions and metabolic pathways of key metabolites in the human body (58). A study has shown that acupuncture GB34 (Yanglingquan) and ST36 (Zusanli) can significantly reduce the number of pro-inflammatory pathogens such as bacteroides, and inhibit the activation and apoptosis of glial cells, thus reducing neuroinflammation (59). Chen et al. (60) found that acupuncture can prevent and weaken the depression-like behaviors of post-chronic unpredictable mild stress rats, possibly by regulating the nitric oxide/cyclic guanosine monophosphate pathway, thus improving the inflammation of serum, lateral habenular nucleus and liver, and the ecological imbalance of intestinal microflora. Jiang et al. (58) made a preliminary study on acupuncture regulating intestinal flora after stroke from the aspects of intestinal microbial structure, intestinal mucosal barrier, hypothalamus-pituitary–adrenal axis, metabolites and metabolic pathways, inflammatory response, and central neurons. Shi et al. (61) found that acupuncture therapy can improve cognitive impairment mainly by regulating intestinal flora, inhibiting inflammatory and improving metabolism and intestinal barrier. Bao et al. (62) showed that acupuncture treatment three times a week for 12 weeks can significantly reduce Crohn’s disease activity index and C-reactive protein, increase the number of operable taxonomic units of intestinal microflora and the relative abundance of Enterobacter faecalis prausnitzii and Rosella faecalis, and effectively reduce the levels of blood diamine oxidase, lipopolysaccharide and T helper type 1/T helper type 17-related cytokines, thus enhancing the intestinal barrier function.

#### 4.2.4 Brain-immune axis

In this study, we found that the brain-immune axis is also the research hotspot and frontier of acupuncture regulating neuroplasticity pathway. Acupuncture has been proved to regulate immune response, especially neuroimmune response (63). The interaction between nervous system and immune system is an important mechanism for acupuncture to regulate the inflammatory process. Acupuncture regulates the immune response of various cell types through local biological and mechanical alterations, including endothelial cells, neutrophils, macrophages, fibroblasts and mast cells, and may regulate the systemic inflammatory response by participating in the vagus nerve system (64). It is found that acupuncture ST36 (Zusanli) achieves anti-inflammatory effect through vagus nerve activation, toll-like receptor (TLR) 4/nuclear factor-kappaB (NF-kappaB) signal, macrophage polarization, mitogen-activated protein kinase (MAPK) signal pathway and cholinergic anti-inflammatory pathway (65). In acupuncture stimulation, macrophages changed from M1 to the M2 phenotype, and the negative TLR4 regulator peroxisome proliferator-activated receptor-gamma is activated to inhibit the intracellular TLR/myeloid differentiation primary response gene 88 and nucleotide binding oligomerization domain signaling pathways, and the downstream inhibitor of nuclear factor Kappa-B isoform alpha/NF-kappaB and P38 MAPK pathways are subsequently inhibited, followed by suppressed production of inflammatory bodies and proinflammatory mediators (66). Acupuncture plays an important role in the plasticity of inflammatory pain neurons, which inhibits the excessive germination of tyrosine hydroxylase immunoreactive of sympathetic nerve, thus alleviating the pain caused by inflammation (67). In addition, acupuncture also regulates the balance of regulatory T cell population. For example, electroacupuncture stimulates the activation of Treg cells to induce the production of higher levels of interleukin-10, and the number of macrophages and neutrophils decreases, thus inhibiting the expression of pro-inflammatory mediators (interleukin-1β, NOD-like receptor family pyrin domain-containing 3 and tumor necrosis factor *α*), and finally inhibiting inflammation and pain (68).

### 4.3 Challenges in the research field of acupuncture regulating neuroplasticity

#### 4.3.1 The mechanism research is not thorough and comprehensive

Although acupuncture has been found to regulate several signal pathways to affect neural plasticity, the relationship between these signal pathways and the network regulation mechanism are not completely clear. For example, in the process of acupuncture regulating neuroplasticity, how to cooperate between neurotrophic factor-related signal pathways and other signal transduction pathways in cells needs further study. The specific effects of different acupuncture points or stimulation parameters on neuroplasticity are also the research focus of the specific molecular mechanism of acupuncture in the future. By using the techniques of transcriptomics, proteomics and metabolomics (69), the changes of genes, protein and metabolites in nerve tissue before and after acupuncture were comprehensively analyzed, and the potential biomarkers and action targets were excavated, and the molecular mechanism and network regulation mechanism of acupuncture regulating neuroplasticity were deeply revealed to further deepen the mechanism research.

In addition, there is a lack of multi-level integration research in this field. Neuroplasticity is a complex process involving molecules, cells, tissues and the whole. At present, acupuncture research is mostly concentrated in one or several levels, lacking the multi-level integration research from genes, protein, cells to the whole animal model. For example, the effect of acupuncture on the expression of some genes has been studied at the molecular level, but the research on how to further affect neural circuits and brain functions at the cellular and tissue levels is still relatively weak. In the future, we should combine neural tracing technology (70), optogenetics (71), brain imaging technology (such as fMRI and diffusion tensor imaging) (72, 73), and to study the influence of acupuncture on neuroplasticity from the aspects of neural circuits and brain networks. To clarify how acupuncture can regulate the connection and information transmission between neurons, and reshape the abnormal brain function network, then to provide a deeper theoretical basis for acupuncture treatment of nervous system diseases.

#### 4.3.2 Defects in clinical research

Our research has found that animal experiments are mostly used in this field at present. However, the results of animal experiments (such as rat model) are difficult to be directly applied to clinic, and how to solve the problem of clinical transformation is still a challenge. From a global perspective, the research on acupuncture regulating neuroplasticity for scholars is still limited. Some clinical studies on acupuncture treatment of neuroplasticity-related diseases have some problems, such as small sample size, insufficient randomization, lack of control or unreasonable control. This leads to the lack of reliability and persuasiveness of the research results, and it is difficult to accurately evaluate the true efficacy and safety of acupuncture.

For animal studies, the ARRIVE 2.0 guidelines should be followed. The control group must be consistent with the intervention group at baseline (same batch of animals, identical genetic background, and feeding environment). Invasive intervention needs sham operation control group, such as only skin incision without acupuncture. An additional positive control drug (such as a BDNF agonist) can be added to verify the mechanism. Furthermore, a verification mechanism for positive control drugs (such as BDNF agonists) can be added. And the calculation method of sample size needs to be clear to ensure statistical efficiency.

In clinical research, the scientific design of a standardized control group is crucial for improving research quality. Clinical trials need to refer to the guidelines on CONSORT expansion of non-drug treatment clinical trials. It is recommended to set up a sham acupuncture control group to eliminate the placebo effect, such as using non-acupoint shallow needling and non-penetrating needles, and ensure that the video rate and communication intensity of treatment visits are consistent with those of the real acupuncture group. When comparing with other therapies, a positive control group is required, adopting adopt clinically recognized standard therapies, strictly follow the guidelines to standardize medication or operation. Meanwhile, the basis for the control group design should be described in detail, and blinding (such as the objective indicator tester is unaware) and hidden distribution measures should be implemented to reduce bias. In the future, it is necessary to follow the basic principles of clinical research and the above guidelines, strictly design large-sample, multi-center, randomized controlled double-blind research, reasonably set up control groups, adopt scientific randomization methods and allocate hidden measures to improve the reliability and scientificity of research results.

In addition, at present, the evaluation indexes of acupuncture efficacy in clinical research are diverse and lack of uniform standards, and different studies adopt different evaluation scales and detection methods. There are differences in the specificity and sensitivity of these indexes in reflecting the influence of acupuncture on neuroplasticity, which is not conducive to the comparison and popularization of research results. A unified, objective, sensitive and specific evaluation system of acupuncture curative effect was established by comprehensively considering various factors that acupuncture affects neural plasticity, combining clinical symptoms, functional recovery, imaging changes and electrophysiological indexes. To ensure the comparability of results between different studies and provide strong evidence for the clinical popularization and application of acupuncture therapy.

#### 4.3.3 Acupuncture technology is difficult to standardize and individualize

Acupuncture therapy has many attributes, such as acupuncture type, acupuncture frequency, selection of acupoints, and whether it can deqi, which may be the influencing factors of acupuncture on nerve plasticity. Few studies directly examine the influence of these parameters on clinical outcomes (74). A study has discussed the dose-effect relationship between different acupuncture doses (frequency, needle retention time and course of treatment) and clinical efficacy (64). It is found that the best frequency of acupuncture is low frequency (once every 2 days) and medium frequency (once per day), and the best course of treatment is short-term (<14 days). Individual differences in acupuncture efficacy and standardized treatment schemes still need to be verified. In the future, based on big data analysis and artificial intelligence technology, combined with the individual characteristics of patients (including physique, genetic characteristics, illness, course of disease, etc.), a personalized acupuncture treatment model can be established. Formulate the most suitable acupuncture points, techniques, frequency and course of treatment for each patient to achieve accurate treatment and improve the effect of acupuncture treatment.

## 5 Limitations of the study

Although this study is strictly implemented, there are still some limitations to be discussed. First, due to the limitation of bibliometrics software, all bibliometrics data contained in this study are from SCI-E database, and some related literatures may be inevitably omitted. Secondly, this study only includes English articles and reviews, and non-English or other types of published documents are not included in this study. As many literatures on acupuncture trials are published in Chinese, the English - only search may omit significant studies, potentially skewing hotspot mapping and trend analysis. In addition, different countries/regions have different investment in acupuncture technology, which may lead to publication bias. However, through bibliometric analysis, this study presents the research status and trend in the field of acupuncture regulating neuroplasticity. The visual analysis of published literatures is helpful for researchers to quickly understand the hotspots and trends in this field and provide a basis for finding new research directions.

## 6 Conclusion

Bibliometric analysis of acupuncture regulating neuroplasticity provides valuable insights on the current research status and development trend. With the increasing research in this field, cooperation between different countries/regions, institutions and authors should be strengthened. AMPK, BDNF, brain-gut axis and brain-immune axis are the research hotspots and frontiers in this field. In the future, with the improvement of technical level, we should deepen the mechanism research, optimize clinical research and promote the standardization and individualization of acupuncture technology, which is expected to further reveal the scientific connotation of acupuncture regulation plasticity.

## Data Availability

The original contributions presented in the study are included in the article/supplementary material, further inquiries can be directed to the corresponding author.

## References

[1] Axelrod CJ Gordon SP Carlson BA. Integrating neuroplasticity and evolution. Curr Biol. (2023) 33:R288–93. doi: 10.1016/j.cub.2023.03.002, PMID: 37098327

[2] Pickersgill JW Turco CV Ramdeo K Rehsi RS Foglia SD Nelson AJ. The combined influences of exercise, diet and sleep on neuroplasticity. Front Psychol. (2022) 13:831819. doi: 10.3389/fpsyg.2022.831819, PMID: 35558719 PMC9090458

[3] Jung J Lambon Ralph MA. The immediate impact of transcranial magnetic stimulation on brain structure: short-term neuroplasticity following one session of cTBS. NeuroImage. (2021) 240:118375. doi: 10.1016/j.neuroimage.2021.118375, PMID: 34245868 PMC8456691

[4] Cabral DF Bigliassi M Cattaneo G Rundek T Pascual-Leone A Cahalin LP . Exploring the interplay between mechanisms of neuroplasticity and cardiovascular health in aging adults: a multiple linear regression analysis study. Auton Neurosci. (2022) 242:103023. doi: 10.1016/j.autneu.2022.103023, PMID: 36087362 PMC11012134

[5] Seng C Luo W Földy C. Circuit formation in the adult brain. Eur J Neurosci. (2022) 56:4187–213. doi: 10.1111/ejn.15742, PMID: 35724981 PMC9546018

[6] Xu H Luo Y Li Q Zhu H. Acupuncture influences multiple diseases by regulating gut microbiota. Front Cell Infect Microbiol. (2024) 14:1371543. doi: 10.3389/fcimb.2024.1371543, PMID: 39040602 PMC11260648

[7] Tsai AWW D'Alessandro E Brandão S Guerreiro JB Bassetto RM Bandeira JS . Acupuncture in cancer care: a narrative review. Rev Assoc Med Bras. (2024) 70:e2024S101. doi: 10.1590/1806-9282.2024S101, PMID: 38865521 PMC11164288

[8] Yao LL Du X Fu YB Zhang F Zhang T Huang F . Preliminary study on mechanism of acupuncture and moxibustion regulating. J Clin Acupunct Moxibust. (2022) 38:1–5. doi: 10.19917/j.cnki.1005-0779.022182

[9] Peng Y Li N Du X Zhang G Huang S Ma J. Acupuncture combined with mirror therapy for post-stroke dyskinesia: a meta-analysis and systematic review. Medicine. (2024) 103:e38733. doi: 10.1097/MD.0000000000038733, PMID: 38941386 PMC11466092

[10] Fang W Ma X Liu B. Global research progress in antibody-drug conjugates for solid tumors: Bibliometrics and visualized analysis. Hum Vaccin Immunother. (2025) 21:2472493. doi: 10.1080/21645515.2025.2472493, PMID: 40013384 PMC11869778

[11] Qu F Wang G Wen P Liu X Zeng X. Knowledge mapping of immunotherapy for breast cancer: a bibliometric analysis from 2013 to 2022. Hum Vaccin Immunother. (2024) 20:2335728. doi: 10.1080/21645515.2024.2335728, PMID: 38563136 PMC10989689

[12] Miao YD Quan WX Zhang F. The research trends in total mesorectal excision in the past twenty years: a bibliometric analysis - correspondence. Int J Surg. (2024) 110:625–7. doi: 10.1097/JS9.0000000000000831, PMID: 37924490 PMC10793780

[13] Zou M Zhang W Xu Y Zhu Y. Relationship between COPD and GERD: a bibliometrics analysis. Int J Chron Obstruct Pulmon Dis. (2022) 17:3045–59. doi: 10.2147/COPD.S391878, PMID: 36510485 PMC9738194

[14] Maiese K. The metabolic basis for nervous system dysfunction in Alzheimer's disease, Parkinson's disease, and Huntington's disease. Curr Neurovasc Res. (2023) 20:314–33. doi: 10.2174/1567202620666230721122957, PMID: 37488757 PMC10528135

[15] Zheng S Wang Y Geng J Liu X Huo L. Global trends in research on MOG antibody-associated disease: Bibliometrics and visualization analysis. Front Immunol. (2024) 15:1278867. doi: 10.3389/fimmu.2024.1278867, PMID: 38370410 PMC10869486

[16] Zhao Y Xue J. Bibliometric analysis of laryngeal cancer treatment literature (2003-2023). Heliyon. (2024) 11:e40832. doi: 10.1016/j.heliyon.2024.e40832 39811326 PMC11730226

[17] Hu Y Yang R Ni S Song Z. Bibliometric analysis of targeted immunotherapy for osteosarcoma-current knowledge, hotspots and future perspectives. Front Immunol. (2025) 15:1485053. doi: 10.3389/fimmu.2024.1485053, PMID: 39995821 PMC11847827

[18] Xiao LY Wang XR Yang Y Yang JW Cao Y Ma SM . Applications of acupuncture therapy in modulating plasticity of central nervous system. Neuromodulation. (2018) 21:762–76. doi: 10.1111/ner.12724, PMID: 29111577

[19] Zheng Y Qin Z Tsoi B Shen J Zhang ZJ. Electroacupuncture on trigeminal nerve-innervated acupoints ameliorates poststroke cognitive impairment in rats with middle cerebral artery occlusion: involvement of neuroprotection and synaptic plasticity. Neural Plast. (2020) 2020:1–13. doi: 10.1155/2020/8818328, PMID: 32963517 PMC7492933

[20] Wang HL Liu FL Li RQ Wan MY Li JY Shi J . Electroacupuncture improves learning and memory functions in a rat cerebral ischemia/reperfusion injury model through PI3K/Akt signaling pathway activation. Neural Regen Res. (2021) 16:1011–6. doi: 10.4103/1673-5374.300454, PMID: 33269744 PMC8224106

[21] Wang L Yang JW Lin LT Huang J Wang XR Su XT . Acupuncture attenuates inflammation in microglia of vascular dementia rats by inhibiting mi R-93-mediated TLR4/MyD88/NF-κB signaling pathway. Oxidative Med Cell Longev. (2020) 2020:8253904. doi: 10.1155/2020/8253904, PMID: 32850002 PMC7441436

[22] Liu CZ Chen JD Zhang M. Advances on the acupuncture therapies and neuroplasticity. Evid Based Complement Alternat Med. (2018) 2018:7231378. doi: 10.1155/2018/7231378, PMID: 30647763 PMC6311774

[23] Su X Wu Z Mai F Fan ZY Du SJ Qian H . 'Governor vessel-unblocking and mind-regulating' acupuncture therapy ameliorates cognitive dysfunction in a rat model of middle cerebral artery occlusion. Int J Mol Med. (2019) 43:221–32. doi: 10.3892/ijmm.2018.3981, PMID: 30431067 PMC6257833

[24] Wu Z Chen S Wang Y Li F Xu H Li M . Current perspectives and trend of computer-aided drug design: a review and bibliometric analysis. Int J Surg. (2024) 110:3848–78. doi: 10.1097/JS9.0000000000001289, PMID: 38502850 PMC11175770

[25] Pan D Wang J Wang H Wu S Guo J Guo L . Mapping the blueprint of artificial blood vessels research: a bibliometric analysis. Int J Surg. (2025) 111:1014–31. doi: 10.1097/JS9.0000000000001877, PMID: 38913439 PMC11745618

[26] Li D Chen QX Zou W Sun XW Yu XP Dai XH . Acupuncture promotes functional recovery after cerebral hemorrhage by upregulating neurotrophic factor expression. Neural Regen Res. (2020) 15:1510–7. doi: 10.4103/1673-5374.257532, PMID: 31997816 PMC7059575

[27] Zhang B Shi H Cao S Xie L Ren P Wang J . Revealing the magic of acupuncture based on biological mechanisms: a literature review. Biosci Trends. (2022) 16:73–90. doi: 10.5582/bst.2022.01039, PMID: 35153276

[28] Trefts E Shaw RJ. AMPK: restoring metabolic homeostasis over space and time. Mol Cell. (2021) 81:3677–90. doi: 10.1016/j.molcel.2021.08.015, PMID: 34547233 PMC8549486

[29] Spaulding HR Yan Z. AMPK and the adaptation to exercise. Annu Rev Physiol. (2022) 84:209–27. doi: 10.1146/annurev-physiol-060721-095517, PMID: 35143330 PMC8919726

[30] Arabmoazzen S Mirshekar MA. Evaluation of the effects of metformin as adenosine monophosphate-activated protein kinase activator on spatial learning and memory in a rat model of multiple sclerosis disease. Biomed Pharmacother. (2021) 141:111932. doi: 10.1016/j.biopha.2021.111932, PMID: 34323699

[31] Cao Y Yang L Cheng H. Ginkgolide B protects against ischemic stroke via targeting AMPK/PINK1. Front Pharmacol. (2022) 13:941094. doi: 10.3389/fphar.2022.941094, PMID: 35837278 PMC9273931

[32] Muraleedharan R Dasgupta B. AMPK in the brain: its roles in glucose and neural metabolism. FEBS J. (2022) 289:2247–62. doi: 10.1111/febs.16151, PMID: 34355526

[33] Jin H Huang R Li Z Liu M Zhao N Zhang H . Acupuncture improves spatial learning and memory impairment caused by herpes simplex virus type-1 in rats through the p 38 MAPK/CREB pathway. J Physiol Sci. (2024) 74:49. doi: 10.1186/s12576-024-00941-4, PMID: 39363248 PMC11448188

[34] Guo K Lu Y. Acupuncture modulates the AMPK/PGC-1 signaling pathway to facilitate mitochondrial biogenesis and neural recovery in ischemic stroke rats. Front Mol Neurosci. (2024) 17:1388759. doi: 10.3389/fnmol.2024.1388759, PMID: 38813438 PMC11133568

[35] Cui Y Hu C Niu C He M Qiu X Yao Q . Electroacupuncture attenuates spared nerve injury-induced neuropathic pain possibly by promoting the progression of AMPK/mTOR-mediated autophagy in spinal microglia. Ann Transl Med. (2022) 10:1278. doi: 10.21037/atm-22-5273, PMID: 36618785 PMC9816825

[36] Guo F Fu L Lu Z. Effect of electroacupuncture combined with sulforaphane in the treatment of sarcopenia in SAMP8 mice. Iran J Basic Med Sci. (2024) 27:560–6. doi: 10.22038/IJBMS.2024.71345.15509, PMID: 38629101 PMC11017848

[37] Ding Z Gao J Feng Y Wang M Zhao H Wu R . Electroacupuncture ameliorates depression-like behaviors in post-stroke rats via activating AMPK-mediated mitochondrial function. Neuropsychiatr Dis Treat. (2023) 19:2657–71. doi: 10.2147/NDT.S436177, PMID: 38077236 PMC10705535

[38] Lan Y Jing X Zhou Z Rao Y Wang K Qin R . Electroacupuncture ameliorates inflammatory pain through CB2 receptor-dependent activation of the AMPK signaling pathway. Chin Med. (2024) 19:176. doi: 10.1186/s13020-024-01048-z, PMID: 39719630 PMC11667860

[39] Lyu Q Shi LQ Chen HY Lu M Liang XC Ma XD . Electroacupuncture combined with NSCs-Exo alters the response of hippocampal neurons in a chronic unpredictable mild stress paradigm in ovx rats. Life Sci. (2024) 359:123235. doi: 10.1016/j.lfs.2024.123235, PMID: 39528081

[40] Liberona A Jones N Zúñiga K Garrido V Zelada MI Silva H . Brain-derived neurotrophic factor (BDNF) as a predictor of treatment response in schizophrenia and bipolar disorder: a systematic review. Int J Mol Sci. (2024) 25:11204. doi: 10.3390/ijms252011204 39456983 PMC11508575

[41] Shafiee A Jafarabady K Mohammadi I Rajai S. Brain-derived neurotrophic factor (BDNF) levels in panic disorder: a systematic review and meta-analysis. Brain Behav. (2024) 14:e3349. doi: 10.1002/brb3.3349, PMID: 38376041 PMC10757897

[42] Gao K Ayati M Koyuturk M Calabrese JR Ganocy SJ Kaye NM . Protein biomarkers in monocytes and CD4+ lymphocytes for predicting lithium treatment response of bipolar disorder: a feasibility study with tyramine-based signal-amplified flow cytometry. Psychopharmacol Bull. (2022) 52:8–35. 10.64719/pb.4424 PMC8896753 35342205

[43] Merighi A. Brain-derived neurotrophic factor, nociception, and pain. Biomolecules. (2024) 14:539. doi: 10.3390/biom14050539, PMID: 38785946 PMC11118093

[44] Lee B. Neuroprotective effect of acupuncture against single prolonged stress-induced memory impairments and inflammation in rat brain via modulation of brain-derived neurotrophic factor expression. Evid Based Complement Alternat Med. (2022) 2022:4430484. doi: 10.1155/2022/4430484, PMID: 35251208 PMC8890831

[45] Liou KT Garland SN Li QS Sadeghi K Green J Autuori I . Effects of acupuncture versus cognitive behavioral therapy on brain-derived neurotrophic factor in cancer survivors with insomnia: an exploratory analysis. Acupunct Med. (2021) 39:637–45. doi: 10.1177/0964528421999395, PMID: 33752446 PMC9281995

[46] Yang P Chen H Wang T Su H Li J He Y . Electroacupuncture promotes synaptic plasticity in rats with chronic inflammatory pain-related depression by upregulating BDNF/TrkB/CREB signaling pathway. Brain Behav. (2023) 13:e 3310. doi: 10.1002/brb3.3310, PMID: 37948105 PMC10726860

[47] Yang NN Lin LL Li YJ Li HP Cao Y Tan CX . Potential mechanisms and clinical effectiveness of acupuncture in depression. Curr Neuropharmacol. (2022) 20:738–50. doi: 10.2174/1570159X19666210609162809, PMID: 35168522 PMC9878952

[48] Jiang H Chen S Li C Lu N Yue Y Yin Y . The serum protein levels of the tPA-BDNF pathway are implicated in depression and antidepressant treatment. Transl Psychiatry. (2017) 7:e1079. doi: 10.1038/tp.2017.43, PMID: 28375203 PMC5416686

[49] Luo T Tian H Song H Zhao J Liya A Fang Y . Possible involvement of tissue plasminogen activator/brain-derived neurotrophic factor pathway in anti-depressant effects of electroacupuncture in chronic unpredictable mild stress-induced depression in rats. Front Psych. (2020) 11:63. doi: 10.3389/fpsyt.2020.00063, PMID: 32153441 PMC7044269

[50] Zhang K Zhai W Ge X Zhang X Tian W Zhai X. Targeting BDNF with acupuncture: a novel integrated strategy for diabetes and depression comorbidity. Heliyon. (2023) 9:e22798. doi: 10.1016/j.heliyon.2023.e22798, PMID: 38125513 PMC10731078

[51] Kawanokuchi J Takagi K Tanahashi N Yamamoto T Nagaoka N Ishida T . Acupuncture treatment for social defeat stress. Front Behav Neurosci. (2021) 15:685433. doi: 10.3389/fnbeh.2021.685433, PMID: 34393735 PMC8355549

[52] You X Niu L Fu J Ge S Shi J Zhang Y . Bidirectional regulation of the brain-gut-microbiota axis following traumatic brain injury. Neural Regen Res. (2025) 20:2153–68. doi: 10.4103/NRR.NRR-D-24-00088, PMID: 39359076 PMC11759007

[53] Wronka D Karlik A Misiorek JO Przybyl L. What the gut tells the brain-is there a link between microbiota and Huntington's disease? Int J Mol Sci. (2023) 24:4477. doi: 10.3390/ijms24054477, PMID: 36901907 PMC10003333

[54] Reutov VP Sorokina EG. Causal relationship between physiological and pathological processes in the brain and in the gastrointestinal tract: the brain-intestine axis. Biophysics. (2022) 67:972–86. doi: 10.1134/S0006350922060197, PMID: 36883179 PMC9984134

[55] Cryan JF O’Riordan KJ Cowan CSM Sandhu KV Bastiaanssen TFS Boehme M . The microbiota-gut-brain axis. Physiol Rev. (2019) 99:1877–2013. doi: 10.1152/physrev.00018.2018 31460832

[56] Loh JS Mak WQ Tan LKS Ng CX Chan HH Yeow SH . Microbiota-gut-brain axis and its therapeutic applications in neurodegenerative diseases. Signal Transduct Target Ther. (2024) 9:37. doi: 10.1038/s41392-024-01743-1, PMID: 38360862 PMC10869798

[57] Lv Z Liu R Su K Gu Y Fang L Fan Y . Acupuncture ameliorates breast cancer-related fatigue by regulating the gut microbiota-gut-brain axis. Front Endocrinol. (2022) 13:921119. doi: 10.3389/fendo.2022.921119, PMID: 36093113 PMC9449876

[58] Jiang H Deng S Zhang J Chen J Li B Zhu W . Acupuncture treatment for post-stroke depression: intestinal microbiota and its role. Front Neurosci. (2023) 17:1146946. doi: 10.3389/fnins.2023.1146946, PMID: 37025378 PMC10070763

[59] Jang JH Yeom MJ Ahn S Oh JY Ji S Kim TH . Acupuncture inhibits neuroinflammation and gut microbial dysbiosis in a mouse model of Parkinson's disease. Brain Behav Immun. (2020) 89:641–55. doi: 10.1016/j.bbi.2020.08.015, PMID: 32827699

[60] Chen W Chen Y Aslam MS Shen J Tong T Yan S . The effect of acupuncture on lateral habenular nucleus and intestinal microflora in depression model rats. Behav Brain Res. (2023) 455:114627. doi: 10.1016/j.bbr.2023.114627, PMID: 37619770

[61] Shi J Zhang X Chen J Shen R Cui H Wu H. Acupuncture and moxibustion therapy for cognitive impairment: the microbiome-gut-brain axis and its role. Front Neurosci. (2024) 17:1275860. doi: 10.3389/fnins.2023.1275860, PMID: 38274501 PMC10808604

[62] Bao C Wu L Wang D Chen L Jin X Shi Y . Acupuncture improves the symptoms, intestinal microbiota, and inflammation of patients with mild to moderate Crohn's disease: a randomized controlled trial. EClinicalMedicine. (2022) 45:101300. doi: 10.1016/j.eclinm.2022.101300, PMID: 35198926 PMC8850329

[63] Kuang H Zhu X Chen H Tang H Zhao H. The immunomodulatory mechanism of acupuncture treatment for ischemic stroke: research progress, prospects, and future direction. Front Immunol. (2024) 15:1468179. doi: 10.3389/fimmu.2024.1319863 38756772 PMC11096548

[64] Cai X Liu H Zhang ZJ Tang C Huang Y. Editorial: neuro-immune-endocrine mechanism of acupuncture in the treatment for nervous system diseases. Front Neurol. (2024) 15:1449040. doi: 10.3389/fneur.2024.1449040, PMID: 39131053 PMC11310110

[65] Oh JE Kim SN. Anti-inflammatory effects of acupuncture at ST36 point: a literature review in animal studies. Front Immunol. (2022) 12:813748. doi: 10.3389/fimmu.2021.813748, PMID: 35095910 PMC8790576

[66] Li N Guo Y Gong Y Zhang Y Fan W Yao K . The anti-inflammatory actions and mechanisms of acupuncture from acupoint to target organs via neuro-immune regulation. J Inflamm Res. (2021) 14:7191–224. doi: 10.2147/JIR.S341581, PMID: 34992414 PMC8710088

[67] Zhang Q Zhou M Huo M Si Y Zhang Y Fang Y . Mechanisms of acupuncture-electroacupuncture on inflammatory pain. Mol Pain. (2023) 19:17448069231202882. doi: 10.1177/17448069231202882, PMID: 37678839 PMC10515556

[68] Yu ML Wei RD Zhang T Wang JM Cheng Y Qin FF . Electroacupuncture relieves pain and attenuates inflammation progression through inducing IL-10 production in CFA-induced mice. Inflammation. (2020) 43:1233–45. doi: 10.1007/s10753-020-01203-2, PMID: 32198725

[69] Yin ZH Bao QN Li YQ Liu YW Wang ZQ Ye F . Discovery of the microbiota-gut-brain axis mechanisms of acupuncture for amnestic mild cognitive impairment based on multi-omics analyses: a pilot study. Complement Ther Med. (2025) 88:103118. doi: 10.1016/j.ctim.2024.103118, PMID: 39667708

[70] Xu D Zou L Zhang W Liao J Wang J Cui J . Comparison of sensory and motor innervation between the acupoints LR3 and LR8 in the rat with regional anatomy and neural tract tracing. Front Integr Neurosci. (2021) 15:728747. doi: 10.3389/fnint.2021.728747, PMID: 34539358 PMC8445157

[71] Ye Q Yuan S Yao L Dai Y Deng B Hu J . Participation of the nucleus tractus solitarius in the therapeutic effect of electroacupuncture on post-stroke dysphagia through the primary motor cortex. CNS Neurosci Ther. (2024) 30:e14442. doi: 10.1111/cns.14442, PMID: 37665118 PMC10916452

[72] Li B Deng S Sang B Zhu W Zhuo B Zhang M . Revealing the neuroimaging mechanism of acupuncture for poststroke aphasia: a systematic review. Neural Plast. (2022) 2022:5635596. doi: 10.1155/2022/5635596, PMID: 35494482 PMC9050322

[73] Liu W Ge W Zhao Q Fan X Li Y Jia H . The neural plasticity and efficacy of acupuncture for post-stroke dysphagia: protocol for a randomized controlled trial with fMRI and DTI. BMC Complement Med Ther. (2024) 24:357. doi: 10.1186/s12906-024-04657-1, PMID: 39367391 PMC11451215

[74] Smith CA Armour M Lee MS Wang LQ Hay PJ. Acupuncture for depression. Cochrane Database Syst Rev. (2018) 2018:CD004046. doi: 10.1002/14651858.CD004046.pub4, PMID: 29502347 PMC6494180

